# Investigation of radiomics models for predicting biochemical recurrence of advanced prostate cancer on pretreatment MR ADC maps based on automatic image segmentation

**DOI:** 10.1002/acm2.14244

**Published:** 2023-12-26

**Authors:** Huihui Wang, Kexin Wang, Shuai Ma, Ge Gao, Xiaoying Wang

**Affiliations:** ^1^ Department of Radiology Peking University First Hospital Beijing China; ^2^ School of Basic Medical Sciences Capital Medical University Beijing China

**Keywords:** apparent diffusion coefficient, biochemical recurrence, prostate cancer, radiomics

## Abstract

**Objectives:**

To develop radiomics models based on automatic segmentation of the pretreatment apparent diffusion coefficient (ADC) maps for predicting the biochemical recurrence (BCR) of advanced prostate cancer (PCa).

**Methods:**

A total of 100 cases with pathologically confirmed PCa were retrospectively included in this study. These cases were randomly divided into training (*n* = 70) and test (*n* = 30) datasets. Two predictive models were constructed based on the combination of age, prostate specific antigen (PSA) level, Gleason score, and clinical staging before therapy and the prostate area (Model_1) or PCa area (Model_2). Another two predictive models were constructed based on only prostate area (Model_3) or PCa area (Model_4). The area under the receiver operating characteristic curve (ROC AUC) and precision‐recall (PR) curve analysis were used to analyze the models’ performance.

**Results:**

Sixty‐five patients without BCR (BCR−) and 35 patients with BCR (BCR+) were confirmed. The age, PSA, volume, diameter and ADC value of the prostate and PCa were not significantly different between the BCR− and BCR+ groups or between the training and test datasets (all *p *> 0.05). The AUCs were 0.637 (95% CI: 0.434–0.838), 0.841 (95% CI: 0.695–0.940), 0.840 (95% CI: 0.698–0.983), and 0.808 (95% CI: 0.627–0.988) for Model_1 to Model_4 in the test dataset without significant difference. The 95% bootstrap confidence intervals for the areas under the PR curve of the four models were not statistically different.

**Conclusion:**

The radiomics models based on automatically segmented prostate and PCa areas on the pretreatment ADC maps developed in our study can be promising in predicting BCR of advanced PCa.

## 1 INTRODUCTION

It is estimated that about 1.4 million new cases of prostate cancer (PCa) and 375,000 related deaths occurred in 2020.^1^ The management approach is usually personalized and complex, due to both health care and socioeconomic viewpoints. Many patients experienced biochemical recurrence (BCR) during treatment. Among patients undergoing radical prostatectomy (RP) or radiotherapy (RT), 27% and 53% of them have a rising PSA (PSA recurrence). Androgen deprivation therapy will inevitably develop into castration resistance.^2^ So early detection BCR and intervention are essential, in order to improve survival time, maintain a good living quality and avoid the possible complications derived from disease progression and spread.

Radiomics is a new frontier of medicine. Its basis is to extract quantitative features (called radiomics features) from radiology images that cannot be observed by radiologists, and use these data to create a clinical decision support system.^3^ Many studies were devoted to exploring the pretreatment MRI features to predict the recurrence of PCa and results were encouraging.^4^, ^5^, ^6^, ^7^, ^8^ However, these research objects were mainly localized PCa treated with RP and/or RT. In actual clinical work, many cancers are diagnosed in advanced stages.^9^ Considering factors such as the disease status, the patients’ intentions and social economics, many patients may only receive hormonal therapy (HT). But these patients were rarely included in previous studies. In addition, the past studies mostly used manually annotated areas of interest by experts to build radiomics models, which is labor‐intensive and hinders the clinical application of the models.

Thus, our study was aimed to develop radiomics models with automatically segmented regions of interest (ROIs) to predict the probability of BCR occurrence in advanced PCa patients treated with RT and/or HT, or in combination with other systemic therapy.

## 2 MATERIALS AND METHODS

### 2.1 Data enrollment

This retrospective study was approved by the local Institutional Review Board and the requirement for written consent was waived.

In our institution, MR images from 2232 patients suspected of PCa between 2016 and 2020 were retrospectively collected. The inclusion criteria were as follows: (a) Pretreatment MR images were available in the Picture Archiving and Communication Systems (PACS). (b) Patients who received RT, HT, or combination as the initial treatment. (c) Patients followed up at least every three months during the first year and every 6 months in the second year. (d) The clinical data, such as age, PSA, and treatment records, were available. (e) Patients followed for at least 2 years and could be documented as BCR (BCR+) or non BCR (BCR−).  BCR was defined as any PSA increase >2 ng/mL compared to the PSA nadir value for men who underwent RT with or without HT. For patients only received HT, BCR was define as castrate serum testosterone <1.7 nmol/L with either three consecutive increases in PSA at least one week apart resulting in two times exceeding the nadir by 50% and a PSA >2 ng/mL or radiological progression in appearance of new lesions.^8^ The exclusion criteria were as followings: (a) Patients who received any treatment before their initial MRI examination. (b) Patients who underwent RP as first‐line treatment. (c) Patients’ following time less than two years. (d) Insufficient clinical data to evaluate the presence or absence of BCR.

**FIGURE 1:**
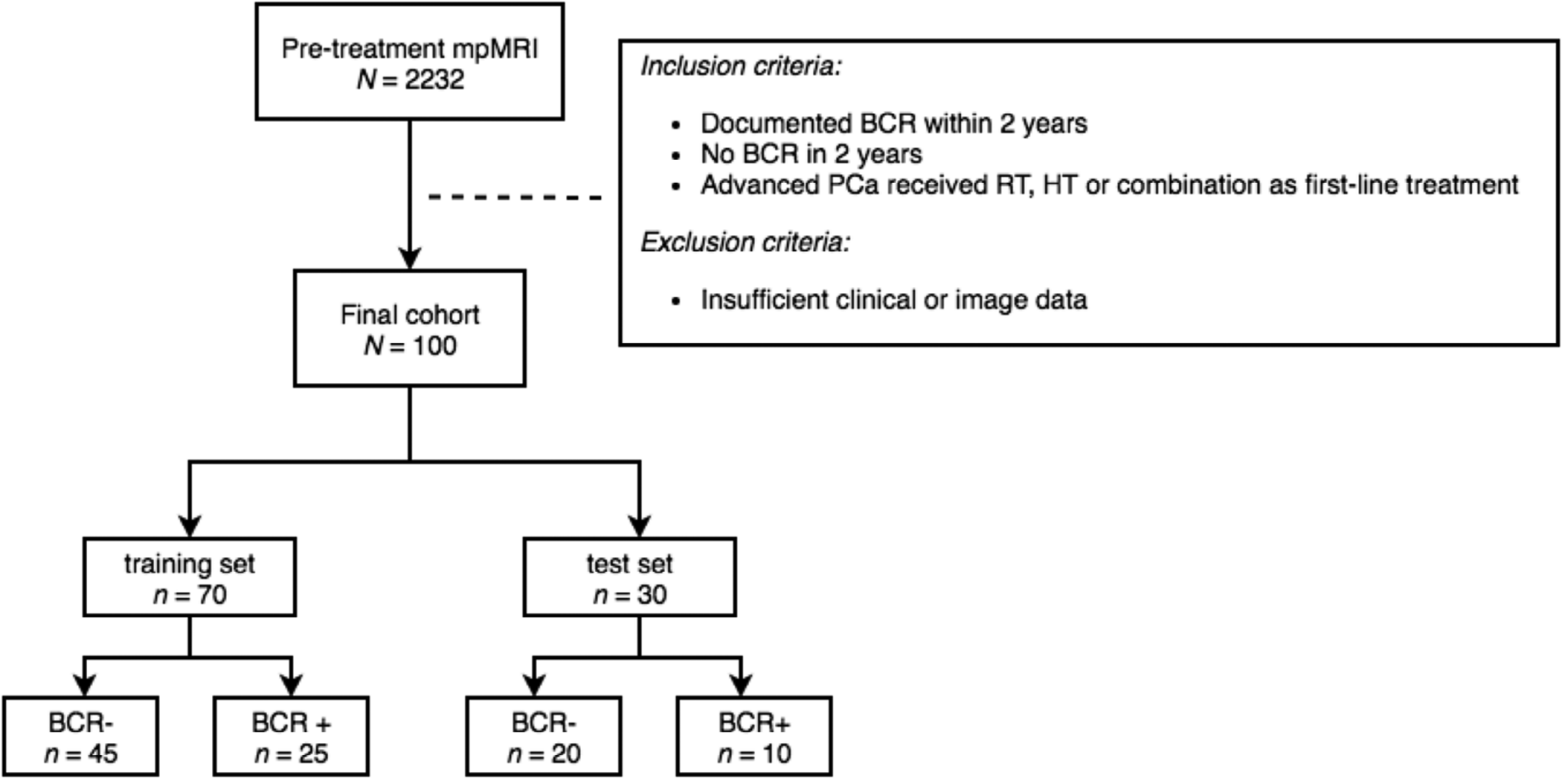
Flow chart of patient enrollment. mpMRI, multiparametric magnetic resonance imaging; BCR, biochemical recurrence; PCa, prostate cancer; RT, radiotherapy; HT, hormonal therapy.

Finally, a total of 100 consecutive patients were included in this study, consisting of 65 BCR− and 35 BCR+. Of these patients, 6 patients only received RT (including 5 BCR− and 1 BCR+, BCR time 23 months), 46 patients received RT with HT (43 BCR− and 3 BCR+, BCR time 10, 10, and 24 months), 46 patients only received HT (15 BCR− and 31 BCR+, BCR time range 3–23 months), and 2 patients received HT with chemotherapy (both BCR−). The 100 cases were randomly assigned into the training dataset (*n* = 70) and test dataset (*n* = 30) in a ratio of 7 to 3 (Figure 1).

### 2.2 MRI acquisition parameters

Full details about acquisition parameters at seven MR scanners were detailed in Table 1. DWI was acquired using an EPI sequence. Only the apparent diffusion coefficient (ADC) images were used for radiomics analysis. ADC maps were calculated using each corresponding vendor‐specific software. Other sequences, including T1, T2‐weighted images and dynamic contrast enhancement, were acquired at the mean time but not analyzed in the current study. To ensure the quality of pelvic scanning images, patients were requested to take laxatives and consume a low residue diet 1 day before the examination.

**TABLE 1:** Image acquisition protocols of ADC maps at seven MR scanners.

	Scanner A	Scanner B	Scanner C	Scanner D	Scanner E	Scanner F	Scanner G	Scanner H
Training dataset, *n*	12	40	4	2	4	1	6	1
Test dataset, *n*	3	18	3	1	1	0	1	3
Magnetic field (T)	1.5	3.0	1.5	3.0	1.5	3.0	1.5	3.0
TR (ms), Min–Max	5010–6010	2000–3200	3650–5200	3580–4000	3120–3430	4100	2460–3720	2000–4440
TE (ms), Min–Max	53.0	58.5–74.3	61.4–70.2	59.1–63.3	81.9–82.8	70.0	71.9–75.5	63.8–67.7
FOV (cm^2^), Min–Max	20 × 20–22 × 22	16 × 16–24 × 24	26 × 26	30 × 30–30 × 30	24 × 24–24 × 24	26 × 26	27 × 27–31 × 31	23 × 23–26 × 26
Section thickness (mm), Min–Max	4.0–4.5	3.0–5.0	4.0–4.0	4.0–5.0	4.0–5.0	4.0–4.0	5.0–5.0	4.0–5.0
Slice spacing (mm)	0	0	0	0	0	0	0	0
Matrix, Min–Max	96 × 96–96 × 96	256 × 256–256 × 256	128 × 128	256 × 256–256 × 256	288 × 288–320 × 320	260 × 260	240 × 240–320 × 320	192 × 192–240 × 240
*b* values, (s/mm^2^), Min–Max	1400	1200–1400	800	800	1400	1000	800–1400	1000–1400

Scanner A: Aera, Siemens Healthcare, Erlangen, Germany. Scanner B: Discovery HD 750, Ge Healthcare, Milwaukee, WI, USA. Scanner C: Signa Excite, GE Healthcare, Milwaukee, WI, USA. Scanner D: Signa Excite, GE Healthcare, Milwaukee, WI, USA. Scanner E: Multiva, Philips Healthcare, Best, The Netherlands. Scanner F: Achieva TX, Philips Healthcare, Best, The Netherlands. Scanner G: Multiva, Philips Healthcare, Best, The Netherlands, different from Scanner E. Scanner H: Signa Excite, GE Healthcare, Milwaukee, WI, USA, the same as Scanner D.

### 2.3 Region of interest

**FIGURE 2:**
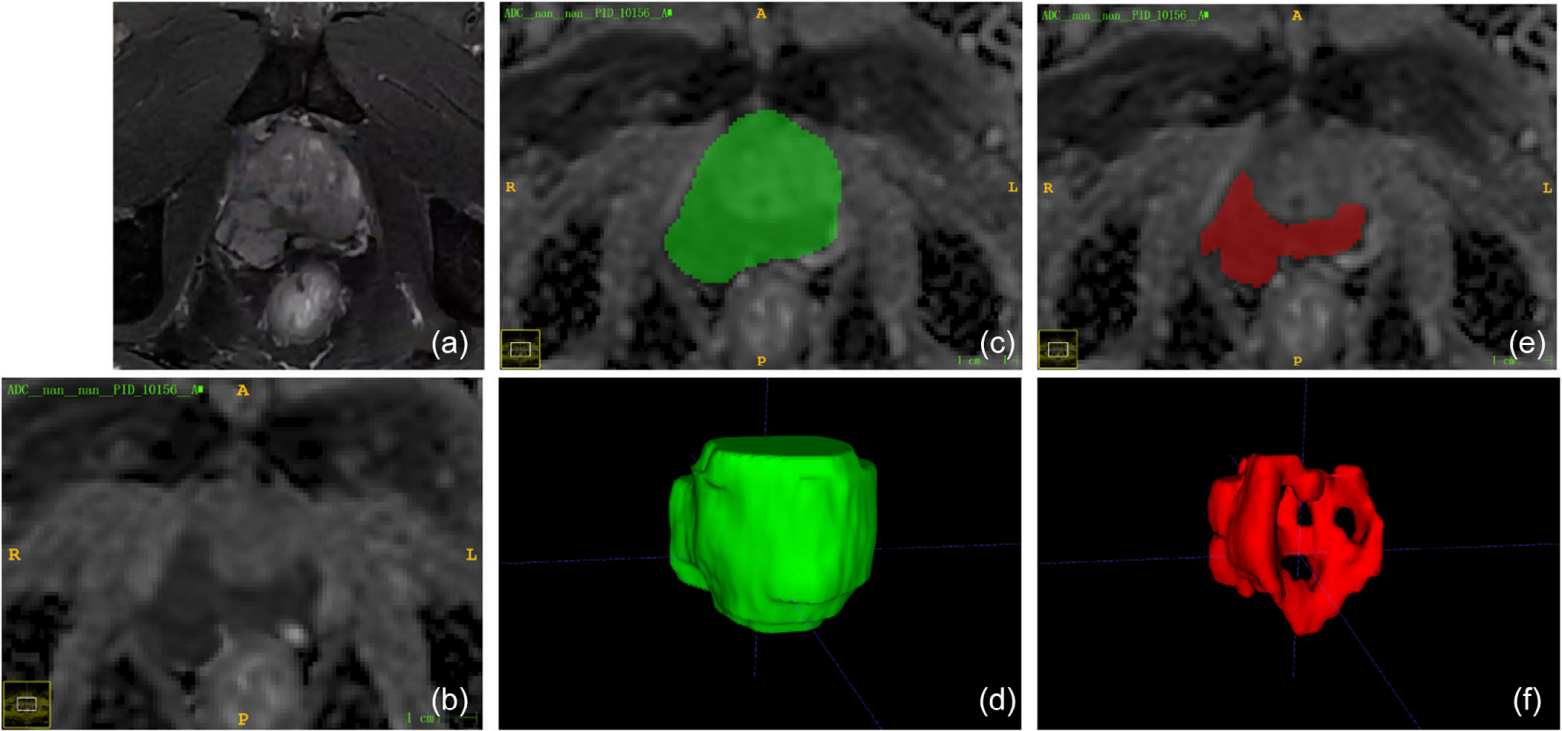
An example of the AI model's automated ROI segmentation. All images in this figure are from the same patient. (a) depicts T2WI, which serves as a reference to reveal the prostate structure. (b) represents the ADC map at the same slice level. (c) displays the prostate region segmented by the AI model, while (d) shows a 3D visualization of the segmented prostate region. (e) represents the suspicious prostate cancer lesions segmented by the AI model within the prostate region, displayed in a single slice. Finally, (f) presents a 3D visualization of the suspicious prostate cancer lesion region.

The areas of the prostate gland and PCa were predicted by priorly trained models (Table S1) on the ADC maps (Figure 2). The workflow of this AI model is as follows: First, the patient's mpMRI images are input into the AI model, which automatically selects the ADC and DWI images. Second, the AI model segments the prostate region from the ADC and DWI images. Third, within the segmented prostate region, the AI model further segments the suspicious areas that are likely to be cancers. The automatically segmented results mentioned above were used in the model development for this study.^10^ If multiple PCa foci were segmented, the largest one was automatically taken as the ROI.

### 2.4 Radiomics modeling

The enrolled data were randomly assigned into two datasets in a ratio of 7:3 (training set: *n* = 70; test set: *n *= 30) (Figure 1). Two predictive models were constructed based on the combination of the age, serum PSA level, Gleason score, and clinical staging before therapy and the prostate area (Model_1) or PCa area (Model_2). The Gleason score from biopsy pathology was categorized into five groups according to the International Society of Urological Pathology (ISUP) classification. The clinical staging was divided into three groups: TxN0M0, TxN1M0, and TxNxM1. Another two predictive models were constructed only based on the automatic prostate area (Model_3) or PCa area (Model_4) (Figure 2).

### 2.5 Preprocess of the images

Since the MR image patterns may differ between different scanners, we tried to reduce the confounding effect by normalizing the image before feature calculation. Three types of images were analyzed, including the (a) “Original Images,” indicating unfiltered images, (b) “LoG Images,” indicating the images filtered with the Laplacian of Gaussian filter, and (c) “Wavelet Images,” indicating the original images underwent a three‐dimensional (i.e., x, y, and z directions) wavelet transformation through the PyWavelet package. Each image was filtered by a high band‐pass filter or low band‐pass in the three directions, resulting in eight combinations of different decompositions: LLH, LHL, HLL, LHH, HHL, HLH, HHH, and LLL (H means high, L means low).

### 2.6 Training and testing the models

Features were extracted from ROIs on the ADC maps using the Pyradiomics package of Python.^11^ Four predictive models were constructed and tested using an established pipeline,^12^, ^13^, ^14^ including three steps: (1) radiomics features extraction, (2) radiomics models development, and (3) predictive performance inspection (Table S2).

### 2.7 Statistical analysis

IBM SPSS^®^ 20.0 (www.ibm.com), MedCalc 20.014 (www.medcalc.org) and R 3.5.1 (www.r‐project.org) were used for statistical analysis.

A Mann–Whitney *U* test or chi‐square test was used to assess differences of clinical characteristics between the training and test cohorts, BCR− and BCR+ groups. The receiver operating characteristic (ROC) curve analysis was employed to estimate the area under the curve (AUC) value of the models. Delong test was used to compare the difference between the AUCs. The precision‐recall (PR) curve analysis was also used to analyze the models’ performance and the 95% bootstrap confidence interval (BC_a_) was used to compare the difference between areas of the PR curves. The level of statistical significance was set at *p* < 0.05.

## 3 RESULTS

### 3.1 Clinical characteristics of the datasets

There were no significant differences in age, PSA, Gleason score, clinical staging, the volume and diameter of prostate gland and tumor, mean ADC value, MR scanner and magnetic field between the training and test datasets (all *p* > 0.05, Table 2). With the exception of clinical staging (*p* = 0.001), the aforementioned clinical characteristics did not exhibit significant differences between the BCR− and BCR+ groups (Table 3). BCR+ rates were 35.7% (25/70) in the training cohort and 33.3% (10/30) in the test cohort, with no significant difference between two cohorts (*χ^2^* = 0.052, *p *= 0.820).

**TABLE 2:** Clinical characteristics of the training and test datasets.

	Level	Train (*n* = 70)	Test (*n* = 30)	*p* value
Age (median [IQR])		74.50 [65.00, 79.00]	73.00 [65.00, 77.25]	0.085
tPSA (median [IQR])		47.25 [19.39, 100.00]	54.20 [19.05, 100.00]	0.566
ISUP	1	2 (2.9%)	1 (3.3%)	0.777
	2	5 (7.1%)	4 (13.3%)	
	3	9 (12.9%)	2 (6.7%)	
	4	12 (17.1%)	6 (20.0%)	
	5	42 (60.0%)	17 (56.7%)	
Clinical staging	TxN0M0	23 (32.9%)	8 (26.7%)	0.260
	TxN1M0	24 (34.3%)	7 (23.3%)	
	TxNxM1	23 (32.9%)	15 (50.0%)	
Prostate volume (median [IQR])		50.61 [36.44, 68.41]	53.16 [37.35, 69.59]	0.172
Prostate ADC (mean (SD))		1.11 (0.19)	1.09 (0.20)	0.342
PCa volume (median [IQR])		15.32 [2.98, 29.90]	15.32 [3.04, 32.82]	0.877
PCa ADC (mean (SD))		0.78 (0.14)	0.78 (0.14)	0.454
Prostate Z (median [IQR])		5.80 [5.22, 6.50]	5.91 [5.23, 6.80]	0.120
PCa Z (mean (SD))		4.90 (1.90)	4.89 (1.92)	0.937
Station Name (%)	Scanner A	12 (17.1)	3(10.0)	0.499
	Scanner B	40 (57.1)	18 (60.0)	
	Scanner C	4 (5.7)	3 (10.0)	
	Scanner D	2 (2.9)	1 (3.3)	
	Scanner E	4 (5.7)	1 (3.3)	
	Scanner F	1 (1.4)	0 (0.0)	
	Scanner G	6 (8.6)	1 (3.3)	
	Scanner H	1 (1.4)	3 (10.0)	
Magnetic Field (%)	1.5T	26 (37.1)	8 (26.7)	0.434
	3.0T	44 (62.9)	22 (73.3)	

**TABLE 3:** Clinical characteristics of the BCR− and BCR+ datasets.

	level	Overall (*n* = 100)	BCR− (*n *= 65)	BCR+ (*n* = 35)	*p* value
Age (median [IQR])		73.00 [65.00, 77.25]	71.00 [65.00, 77.00]	74.00 [66.00, 79.50]	0.354
tPSA (median [IQR])		54.20 [19.05, 100.00]	44.18 [16.46, 98.13]	75.16 [24.87, 100.00]	0.064
PSAD (median [IQR])		1.02 [0.40, 2.89]	0.87 [0.40, 2.09]	1.27 [0.46, 9.86]	0.075
ISUP	1	3 (3.0%)	1 (1.5%)	2 (5.7%)	0.082
	2	9 (9.0%)	9 (13.8%)	0 (0%)	
	3	11 (11.0%)	8 (12.3%)	3 (8.6%)	
	4	18 (18.0%)	13 (20.0%)	5 (14.3%)	
	5	59 (59.0%)	34 (52.3%)	25 (71.4%)	
Clinical staging	TxN0M0	31 (31.0%)	27 (41.5%)	4 (11.4%)	0.001
	TxN1M0	31 (31.0%)	21 (32.3%)	10 (28.6%)	
	TxNxM1	38 (38.0%)	17 (26.2%)	21 (60.0%)	
Prostate volume (median [IQR])		53.16 [37.35, 69.59]	46.46 [34.95, 68.23]	59.21 [43.72, 83.25]	0.067
Prostate ADC (mean (SD))		1.09 (0.20)	1.12 (0.20)	1.04 (0.20)	0.070
PCa volume (median [IQR])		15.32 [3.04, 32.82]	12.53 [2.39, 28.96]	22.13 [4.86, 37.64]	0.059
PCa ADC (mean (SD))		0.78 (0.14)	0.79 (0.14)	0.78 (0.14)	0.815
Prostate diameter (median [IQR])		5.91 [5.23, 6.80]	5.88 [5.06, 6.51]	6.18 [5.54, 7.31]	0.088
PCa diameter (mean (SD))		4.89 (1.92)	4.66 (1.86)	5.31 (1.99)	0.111
Station Name (%)	Scanner A	15 (16.1)	9 (15.3)	6 (17.6)	0.757
	Scanner B	58 (62.4)	37 (62.7)	21 (61.8)	
	Scanner C	7 (7.5)	5 (8.5)	2 (5.9)	
	Scanner D	3 (3.2)	3 (5.1)	0 (0.0)	
	Scanner E	5 (5.4)	3 (5.1)	2 (5.9)	
	Scanner F	1 (1.1)	0 (0.0)	1 (2.9)	
	Scanner G	0 (0.0)	0 (0.0)	0 (0.0)	
	Scanner H	4 (4.3)	2 (3.4)	2 (5.9)	
Magnetic field (%)	1.5T	34 (34.0)	23 (35.4)	11 (31.4)	0.859
	3.0T	66 (66.0)	42 (64.6)	24 (68.6)	

### 3.2 Model evaluation

The features selected from the four models are presented in the supplementary material (Table S3), as well as the parameters of these models (Table S4).

**FIGURE 3:**
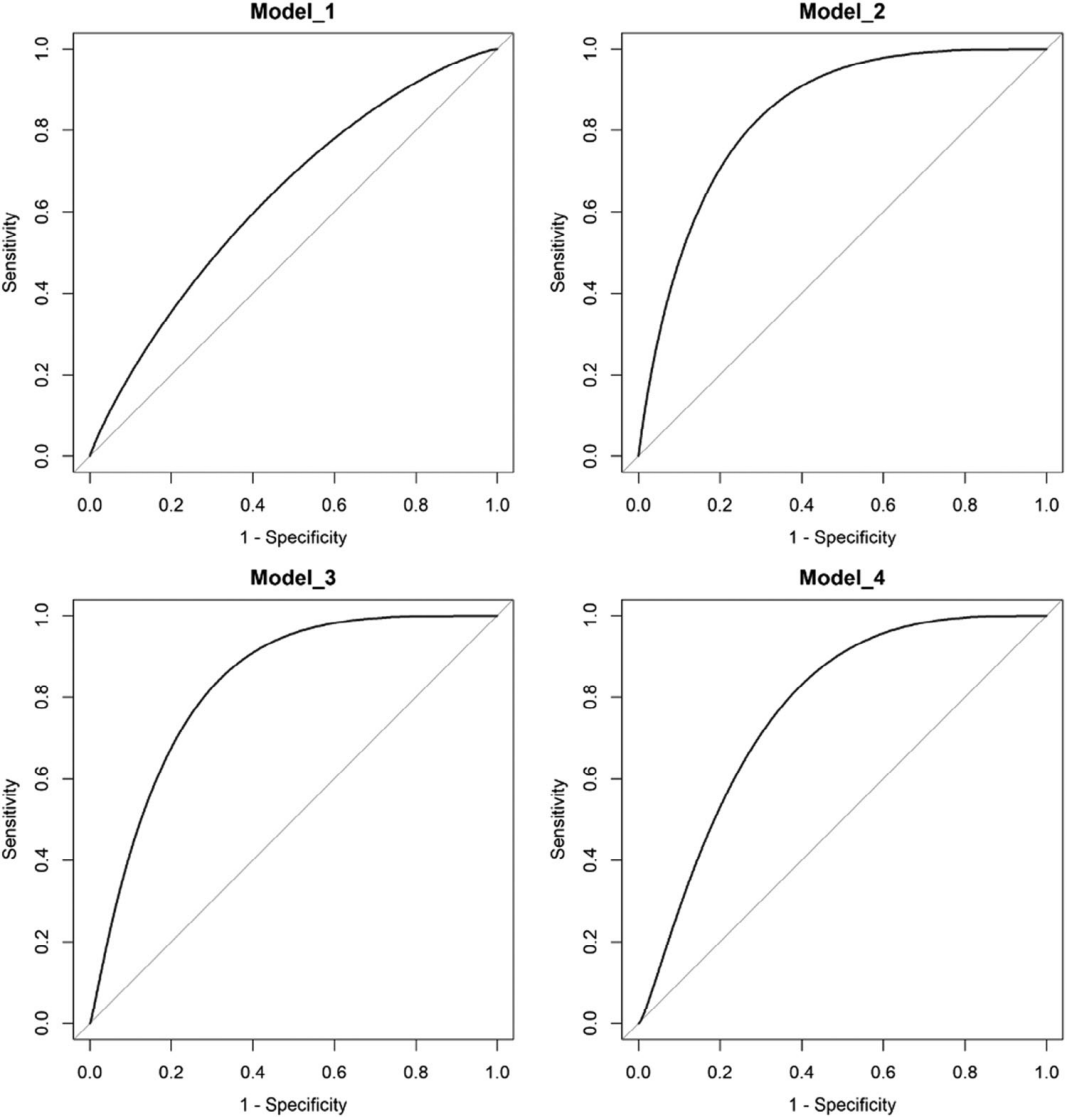
ROC curves for the prediction of biochemical recurrence using the four models for the test dataset.

**FIGURE 4:**
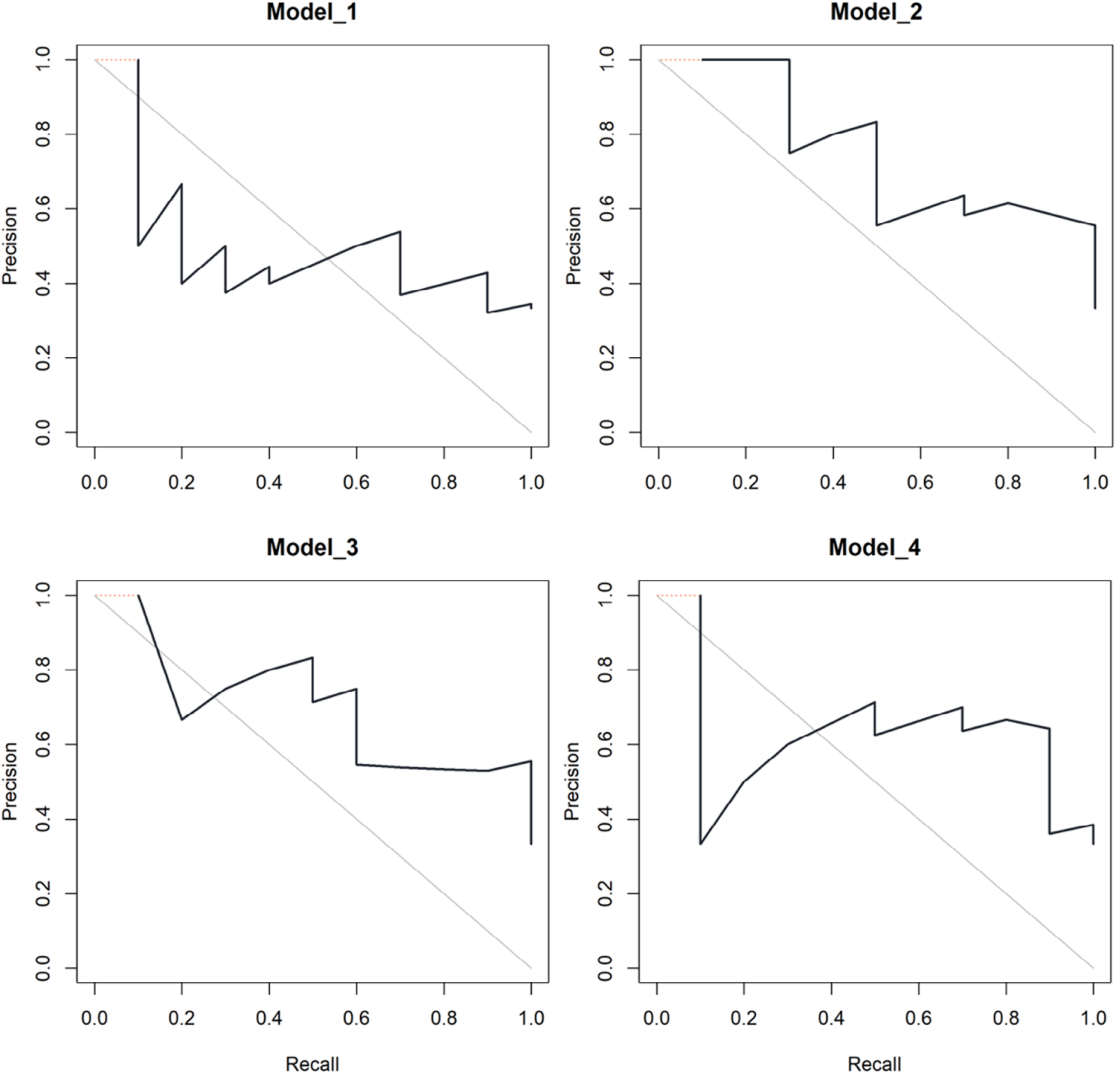
The precision‐recall curves for the prediction of biochemical recurrence using the four models in test dataset.

For the prediction of the PCa BCR, all models were found to perform well and the AUCs were 0.770 (95% CI: 0.527–1.000), 0.793 (95% CI: 0.604–0.981), 0.840 (95% CI: 0.698–0.983), and 0.808 (95% CI: 0.627–0.988) for Model_1 to Model_4 in the test datasets (Figure 3). DeLong test showed no significant difference between each model (all *p *> 0.05, Table 4). The 95% BC_a_ bootstrap confidence interval for the PR area difference included 0 between each model (all *p *> 0.05, Figure 4).

**TABLE 4:** Comparison the AUCs of different models for the test dataset.

	Model_1	Model_2	Model_3
Model_2	0.076	/	/
Model_3	0.153	0.862	/
Model_4	0.152	0.597	0.769

## 4 DISCUSSION

In this present study, we proposed four predictive models with automatic segmentation showing relatively good accuracy in the prediction of BCR for advanced PCa from pretreatment ADC maps. The results of this study are similar to previous studies in demonstrating the predictive efficiency of the radiomics models for BCR^4^, ^5^, ^6^, ^7^, ^8^, ^15^ and differ from previous studies in three ways: (1) the ADC data source is heterogeneous, (2) the ROI of the model is automatically segmented, and (3) our study focuses on advanced PCa patients with non‐surgical treatment.

In clinical practice, the DWI images are actually influenced by various factors such as T1, T2, and even capillary perfusion, resulting in DWI images not presenting as ideal.^16^ To avoid the interference of T2 effect on DWI image interpretation, it is necessary to generate ADC parameter maps. By utilizing MR post‐processing software, it is easy to generate ADC maps, which can remove T2 interference while preserving the inherent contrast features of DWI images. In addition to the interference of T2 shine‐effect on DWI images, the other factor that needs to be considered is the influence of perfusion effect. Each pixel collected on the DWI image actually contains component information of different diffusion degrees, and the measured ADC value is actually a reflection of the comprehensive diffusion information. According to the IVIM theory, when the *b* value is low, it reflects the tissue perfusion information, that is, the diffusion weighted signal mainly reflects the molecular diffusion information of the free diffusion of blood in the capillaries. Using a higher *b* value can reduce the impact of the perfusion effect. That is why most of *b* values in our study were 1400 s/mm^2^ and a small portion were 800 s/mm^2^ applied earlier.

In previous studies, the establishment of radiomics models required the same MR device, or even the same acquisition parameters.^4^, ^15^ The strict requirements of the data source lead to the models generalization difficulty. The heterogenous ADC acquisition parameters were used in our study, mainly due to the seven different MR scanners, with *b* values ranging from 800 to 1400 s/mm^2^. Besides, the development of MR technology and clinical experience promotes continuous updating of scan parameters. Our results showed that promising predictive models could also be obtained using inhomogeneous data, probably due to the characteristics of the ADC maps. Previous phantom studies showed there was a good agreement of the ADC values across different sites, different vendors and different protocols.^17^, ^18^ This phenomenon may suggest that the ADC image features of the lesion are consistent across MR devices and protocols. Of course, this present study was not designed to answer this question, which should be explored in a dedicated study.

The priorly trained deep learning models^19^, ^20^ were used in this study and the radiomics features were extracted from the automatically segmented prostate and PCa areas. The predictive models based on the prostate area (Model_1 and Model_3) performed equivalent to the models based on PCa area (Model_2 and Model_4). The enrolled cases were all advanced and most of tumors occupied a large proportion of the prostate gland, so both represented the overall characteristics of the tumors. This result was similar to previous findings, which showed that extracting MRI radiomics features from the whole prostate gland or PCa area did not change the models’ performance in the prediction of localized PCa BCR. Zhong et al.^4^ demonstrated the potential of mpMRI‐based prostate gland radiomics features to predict the BCR of localized PCa patients after RT in a retrospective study (AUC = 0.73). Shiradkar et al.^6^ promoted a machine‐learning classifier to predict BCR after therapy by extracting the radiomics features in the region of PCa on pretreatment T2‐wighted images and ADC images, with a mean AUC = 0.73 on test dataset. In present study, the predictive efficiency of the four predictive models seemed equivalent or slightly better than the above studies. Besides, the strength of our study is the automatic segmentation of the ROIs compared with above studies. With less manual intervention, it is possible to have more efficient and stable results during application of the models in real clinical practice.

The radiomics‐only models (Model_3 and Model_4) performed slightly better than the radiomics models combined with clinical characteristics (Model_1 and Model_2). Bourbonne et al.^7^ concluded that the radiomics model developed from ADC delineated tumors was predictive in BCR after RP in their study. However, the addition of clinical data to radiomics based models hampered their performance on the test dataset. According to their analysis, the selected population was already at high risk of BCR and very homogeneous in terms of clinical characteristics. Therefore, the stratification based only on clinical characteristics was invalid. Considering the subjects were postoperative patients in their study and the clinical characteristics in our study were not comprehensive, further exploration is needed to identify the robust predictors of BCR to guide post treatment management.

As a common indicator of tumor burden, tumor volume has been proved to be associated with tumor prognosis. In particular, the larger the absolute tumor volume, the higher the risk of BCR.^21^ Meyer et al.^21^ retrospectively analyzed detailed pathological tumor volume in 903 men with pT2 PCa after RP and discovered tumor volume was an independent predictor of BCR. Similarly, Riaz et al.^22^ concluded that tumor size of the dominant lesion measured on pretreatment MRI was associated with BCR in intermediate and high‐risk PCa patients treated with RT, while Gleason score and the presence of extracapsular extension on MRI were the only significant predictors. Xia et al.^8^ retrospectively collected a total of 337 patients who underwent RP and considered that maximum diameter of the index lesion measured on pretreatment MRI was an independent predictor of BCR. Usually PCa is multifocal, so the measurement of tumor volume is complex and time‐consuming and difficult to achieve in clinical practice. Moreover, there may be deviations in the measurement among different radiologists and the same radiologist at different times. For above reasons, the whole prostate gland and the largest PCa lesion, that is, the index lesion, were selected as ROIs by automatic segmentation to maintain consistency in this study. Our study showed no significant differences in tumor diameter and volume between BCR− and BCR+ groups. It may be because the tumor stages in our study were different from those in above studies, and the impact of the primary tumor volume was probably weakened.

Diagnosis of PCa may occur at any stage of disease, and when the diagnosis is made late, PCa is considered to have a poor prognosis. Prognostic factors include the volume or burden of the disease, the time of metastases relative to the initial diagnosis, and patient factors that determine the appropriateness of treatment.^23^ Given the various levels of evidence available at each stage, the treatment options available at any given stage are different.^24^ Urologists play an essential role in helping patients decide the most appropriate treatment. It is crucial to consider the treatment's side effect, economic impact, patient preferences and expectations.^25^, ^26^, ^27^ At present, a variety of agents are available for the treatment of PCa, some of which are considered beneficial in a variety of PCa states. Although the National Comprehensive Cancer Network has developed regularly updated evidence‐based guidelines, evidence still needs to be collected and verified. Considering the above factors, there is no single correct or wrong answer to the treatment of advanced PCa patients in the actual clinical practice, so we still have a long way to go to deliver optimal treatment to those who need it most.^23^ The patients with advanced PCa included in our study lost the opportunity of RP, or their life expectancy was very short and could not benefit from RP. Therefore, RT and HT were used to expect the best prognosis and longer survival time with cancer. In fact, the treatment plan is timely adjusted according to the dynamic changes of PSA and imaging examination, and the clinical work experience of urologists. And we observed that patients treated with only HT in present study had a higher probability of BCR than those treated with only RT or RT combined with HT, which is consistent with the fact that HT was more prone to castrate‐resistant prostate cancer (CRPC).^2^

Certainly, our study has some intrinsic limitations. Firstly, the relatively small cohort in our single institution reduced the statistical power. Though a variety of MR devices and protocols in this study increased repeatability and verifiability of our results, the majority of our data came from two scanners (Scanner A (*n* = 15) and scanner B (*n* = 58) for a total 73 studies). The previous study has shown that the performance in prediction of BCR was higher in 3T compared to 1.5T (AUCs of 0.87 and 0.76, respectively).^7^ In present study, there was no significant difference in field strength between BCR− and BCR+ groups or between the training and test datasets, avoiding the potential impact of field strength on predictive results. Secondly, this study may have selection bias because patients' choice of treatment was affected by urologists and patients related factors, which were not reflected in the data. The treatment options were diverse, and the effect of different options on prognosis was not further analyzed. Thirdly, the other MR sequences, which may have potential impact on prognosis, were not analyzed in this study. Besides, different *b* values were used because of machine produced in different eras and updated guidelines.^28^ The further research would impose stricter restrictions on enrollment conditions. At last, follow‐up time was short, which could not represent a real long‐term prognosis. Data collection will continue in further study to fill these gaps.

## 5 CONCLUSION

Despite the above limitations, radiomics models with an automatic segmentation on the pretreatment MR ADC maps in our study were predictive of advanced PCa BCR. Using the predictive information might facilitate the selection of appropriate individualized treatment regimens.

## AUTHOR CONTRIBUTIONS

*Guarantor of integrity of the entire study*: Xiaoying Wang. *Study concepts and design*: Huihui Wang and Xiaoying Wang. *Literature research*: Huihui Wang and Ge Gao. *Clinical studies*: Shuai Ma and Huihui Wang. *Experimental studies / data analysis*: Kexin Wang, Ge Gao and Huihui Wang. *Statistical analysis*: Kexin Wang, Shuai Ma, Ge Gao and Huihui Wang. *Manuscript preparation*: Huihui Wang and Kexin Wang. *Manuscript editing*: Huihui Wang, Kexin Wang and Xiaoying Wang.

## CONFLICT OF INTEREST STATEMENT

The authors of this manuscript declare no relationships with any companies, whose products or services may be related to the subject matter of the article.

## ETHICAL APPROVAL

This retrospective study was approved by the local Institutional Review Board (2018‐217) and the requirement for written consent was waived.

## Supporting information

Supporting Information

Supporting Information

Supporting Information

Supporting Information
