# Supplementary material for: Investigation of radiomics models for predicting biochemical recurrence of advanced prostate cancer on pretreatment MR ADC maps based on automatic image segmentation
Source: J Appl Clin Med Phys. 2023 Dec 26;25(4):e14244. doi: 10.1002/acm2.14244 (PMC11005965; doi:10.1002/acm2.14244)
Supplement: Supplementary file 1 — Supporting Information [file ACM2-25-e14244-s002.docx]

**Table S1** Extracted radiomics features and their category

| Categories | Features |
| --- | --- |
| First-order (n=18) | 10^th^ Percentile |
|  | 90^th^ Percentile |
|  | Energy |
|  | Entropy |
|  | Interquartile Range |
|  | Kurtosis |
|  | Maximum |
|  | Mean Absolute Deviation |
|  | Mean |
|  | Median |
|  | Minimum |
|  | Range |
|  | Robust Mean Absolute Deviation |
|  | Root Mean Squared |
|  | Skewness |
|  | Total Energy |
|  | Uniformity |
|  | Variance |
| 3D Shape-based (n=14) | Elongation |
|  | Flatness |
|  | Least Axis Length |
|  | Major Axis Length |
|  | Maximum 2D Diameter (Column) |
|  | Maximum 2D Diameter (Row) |
|  | Maximum 2D Diameter (Slice) |
|  | Maximum 3D Diameter |
|  | Mesh Volume |
|  | Minor Axis Length |
|  | Sphericity |
|  | Surface Area |
|  | Surface Volume Ratio |
|  | Voxel Volume |
| Gray Level Co-occurrence | Autocorrelation |
| Matrix (GLCM) (n=24) | Cluster Prominence |
|  | Cluster Shade |
|  | Cluster Tendency |
|  | Contrast |
|  | Correlation |
|  | Difference Average |
|  | Difference Entropy |
|  | Difference Variance |
|  | Inverse Difference (ID) |
|  | Inverse Difference Moment (IDM) |
|  | Inverse Difference Moment Normalized (IDMN) |
|  | Inverse Difference Normalized (IDN) |
|  | Informational Measure of Correlation (IMC) 1 |
|  | Informational Measure of Correlation (IMC) 2 |
|  | Inverse Variance |
|  | Joint Average |
|  | Joint Energy |
|  | Joint Entropy |
|  | Maximal Correlation Coefficient (MCC) |
|  | Maximum Probability |
|  | Sum Average |
|  | Sum Entropy |
|  | Sum of Squares |
| Gray Level Run length | Gray Level Non-Uniformity (GLN) |
| Matrix (GLRLM) (n=16) | Gray Level Non-Uniformity Normalized (GLNN) |
|  | Gray Level Variance (GLV) |
|  | High Gray Level Run Emphasis (HGLRE) |
|  | Long Run Emphasis (LRE) |
|  | Long Run High Gray Level Emphasis (LRHGLE) |
|  | Long Run Low Gray Level Emphasis (LRLGLE) |
|  | Low Gray Level Run Emphasis (LGLRE) |
|  | Run Entropy (RE) |
|  | Run Length Non-Uniformity (RLN) |
|  | Run Length Non-Uniformity Normalized (RLNN) |
|  | Run Percentage (RP) |
|  | Run Variance (RV) |
|  | Short Run Emphasis (SRE) |
|  | Short Run High Gray Level Emphasis (SRHGLE) |
|  | Short Run Low Gray Level Emphasis (SRLGLE) |
| Gray Level Size Zone | Gray Level Non-Uniformity (GLN) |
| Matrix (GLSZM) (n=16) | Gray Level Non-Uniformity Normalized (GLNN) |
|  | Gray Level Variance (GLV) |
|  | High Gray Level Zone Emphasis (HGLZE) |
|  | Large Area Emphasis (LAE) |
|  | Large Area High Gray Level Emphasis (LAHGLE) |
|  | Large Area Low Gray Level Emphasis (LALGLE) |
|  | Low Gray Level Zone Emphasis (LGLZE) |
|  | Size-Zone Non-Uniformity (SZN) |
|  | Size-Zone Non-Uniformity Normalized (SZNN) |
|  | Small Area Emphasis (SAE) |
|  | Small Area High Gray Level Emphasis (SAHGLE) |
|  | Small Area Low Gray Level Emphasis (SALGLE) |
|  | Zone Entropy (ZE) |
|  | Zone Percentage (ZP) |
|  | Zone Variance (ZV) |
| Gray Level Dependence | Dependence Entropy (DE) |
| Matrix (GLDM) (n=14) | Dependence Non-Uniformity (DN) |
|  | Dependence Non-Uniformity Normalized (DNN) |
|  | Dependence Variance (DV) |
|  | Gray Level Non-Uniformity (GLN) |
|  | Gray Level Variance (GLV) |
|  | High Gray Level Emphasis (HGLE) |
|  | Large Dependence Emphasis (LDE) |
|  | Large Dependence High Gray Level Emphasis (LDHGLE) |
|  | Large Dependence Low Gray Level Emphasis (LDLGLE) |
|  | Low Gray Level Emphasis (LGLE) |
|  | Small Dependence Emphasis (SDE) |
|  | Small Dependence High Gray Level Emphasis(SDHGLE) |
|  | Small Dependence Low Gray Level Emphasis (SDLGLE) |
