# Supplementary material for: Investigation of radiomics models for predicting biochemical recurrence of advanced prostate cancer on pretreatment MR ADC maps based on automatic image segmentation
Source: J Appl Clin Med Phys. 2023 Dec 26;25(4):e14244. doi: 10.1002/acm2.14244 (PMC11005965; doi:10.1002/acm2.14244)
Supplement: Supplementary file 2 — Supporting Information [file ACM2-25-e14244-s003.docx]

**Table S2** Available options for each step in the radiomics model development pipeline, including data normalization, dimension reduction, feature selection, and classifier

| Steps | Candidate |
| --- | --- |
| Data Normalization | Min-Max Normalization |
|  | Mean Normalization |
| Feature dimension reduction | Pearson Correlation Coefficient (PCC) |
|  | Principle Component Analysis (PCA) |
| Feature Selection | Analysis of Variance (ANOVA) |
|  | Recursive Feature Elimination (RFE) |
|  | Relief |
|  | Kruskal-Wallis (KW) |
| Classifier | Logistic Regression |
|  | Random Forest  LightGBM  AdaBoost  Support Vector Machine (SVM)  Least Absolute Shrinkage and Selection Operator (LASSO) |
|  | CatBoost |
|  | Decision Tree  eXtremeGradientBoost  GradientBoosting  ExtraTrees |
