# Supplementary material for: Investigation of radiomics models for predicting biochemical recurrence of advanced prostate cancer on pretreatment MR ADC maps based on automatic image segmentation
Source: J Appl Clin Med Phys. 2023 Dec 26;25(4):e14244. doi: 10.1002/acm2.14244 (PMC11005965; doi:10.1002/acm2.14244)
Supplement: Supplementary file 3 — Supporting Information [file ACM2-25-e14244-s001.docx]

**Table S3** Features of the four models

|  | Model_1 | Model_2 | Model_3 | Model_4 |
| --- | --- | --- | --- | --- |
| Clinical features | Gleason score  Clinical staging | Gleason score  Clinical staging | / | / |
| Shape-bases features |  | original_ MajorAxisLength | original_ Maximum2DDiameterSlice | original_ MajorAxisLength |
|  |  | original_ Maximum2DDiameterRow | original_ Maximum3DDiameter | original_ Maximum2DDiameterRow |
|  |  | original_ Maximum2DDiameterSlice |  | original_ Maximum2DDiameterSlice |
|  |  | original_ MinorAxisLength |  | original_ MinorAxisLength |
|  |  | original_ Sphericity |  | original_Sphericity |
|  |  |  |  | original_ SurfaceArea |
| First-order features |  | log-sigma-3-0-mm-3D _Skewness | wavelet-HHH_ Mean | log-sigma-3-0-mm-3D_ Skewness |
|  |  | log-sigma-5-0-mm-3D _Skewness |  | log-sigma-5-0-mm-3D_ Skewness |
|  |  | wavelet-LHH_ Kurtosis |  | wavelet-LHH_Kurtosis |
|  |  |  |  | wavelet-HHH_Kurtosis |
| Texture features | original_glszm_ LargeAreaLowGrayLevelEmphasis | log-sigma-3-0-mm-3D_glcm_ ClusterShade | wavelet-HLH_glcm_ ClusterShade | log-sigma-3-0-mm-3D_glcm_ ClusterShade |
|  | wavelet-LHL_glcm_ClusterProminence | log-sigma-5-0-mm-3D_glcm_ ClusterShade | original_glszm_ LargeAreaLowGrayLevelEmphasis | log-sigma-3-0-mm-3D_glcm_ ClusterTendency |
|  | wavelet-LHL_gldm_ LargeDependenceLowGrayLevelEmphasis | log-sigma-5-0-mm-3D_glszm_ SmallAreaLowGrayLevelEmphasis |  | log-sigma-3-0-mm-3D_glcm_ SumEntropy |
|  | wavelet-HLH_glcm_ ClusterProminence | wavelet-HHH_glcm_ Idmn |  | log-sigma-5-0-mm-3D_glcm_ ClusterShade |
|  | wavelet-LHH_glszm_ LowGrayLevelZoneEmphasis | wavelet-HHH_glszm_ GrayLevelNonUniformityNormalized |  | log-sigma-5-0-mm-3D_glszm_ SmallAreaLowGrayLevelEmphasis |
|  |  | wavelet-HHH_glszm_ ZoneEntropy |  | wavelet-HHL_glszm_ GrayLevelNonUniformityNormalized |
|  |  |  |  | wavelet-HHH_glcm_ Idmn |
|  |  |  |  | wavelet-HHH_glszm_ GrayLevelNonUniformityNormalized |
|  |  |  |  | wavelet-HHH_glszm_ ZoneEntropy |
