# Supplementary material for: Investigation of radiomics models for predicting biochemical recurrence of advanced prostate cancer on pretreatment MR ADC maps based on automatic image segmentation
Source: J Appl Clin Med Phys. 2023 Dec 26;25(4):e14244. doi: 10.1002/acm2.14244 (PMC11005965; doi:10.1002/acm2.14244)
Supplement: Supplementary file 4 — Supporting Information [file ACM2-25-e14244-s004.docx]

**Table S4** Radiomics pipeline of the four models

|  | Data Normalization | Feature dimension reduction | Feature Selection | Feature number in the classifier | Type of classifier |
| --- | --- | --- | --- | --- | --- |
| Model_1 | MinMaxNormalizer | PCC | Relief | 6 | eXtremeGradientBoost |
| Model_2 | MeanNormalizer | PCC | KW | 14 | ExtraTrees |
| Model_3 | MeadNormalizer | PCC | Relief | 5 | GradientBoosting |
| Model_4 | MinMaxNormalizer | PCC | KW | 19 | ExtraTrees |
